# Impact of immigration on the cost of emergency visits in Barcelona (Spain)

**DOI:** 10.1186/1472-6963-7-9

**Published:** 2007-01-19

**Authors:** Francesc Cots, Xavier Castells, Oscar García, Marta Riu, Aida Felipe, Oriol Vall

**Affiliations:** 1 Health Services Evaluation and Clinical Epidemiology, Department, Institut Municipal d'Assistència Sanitària (IMAS), Barcelona, Spain; 2 Health Services Research Unit, (IMIM-IMAS), Barcelona, Spain; 3 Paediatric Service, Institut Municipal d'Assistència Sanitària (IMAS), Barcelona, Spain; 4 Childhood and Environment Research Unit, Institut Municipal d'Investigació Mèdica (IMIM-IMAS), Barcelona, Spain; 5 Departament de Pediatria, Obstetrícia i Ginecologia i Medicina Preventiva, Universitat Autònoma de Barcelona, Barcelona, Spain

## Abstract

**Background:**

The impact of immigration on health services utilisation has been analysed by several studies performed in countries with lower levels of immigration than Spain. These studies indicate that health services utilisation is lower among the immigrant population than among the host population and that immigrants tend to use hospital emergency services at the expense of primary care. We aimed to quantify the relative over-utilisation of emergency services in the immigrant population.

**Methods:**

Emergency visits to Hospital del Mar in Barcelona in 2002 and 2003 were analysed. The country of origin, gender, age, discharge-related circumstances (hospital admission, discharge to home, or death), medical specialty, and variable cost related to medical care were registered. Immigrants were grouped into those from high-income countries (IHIC) and those from low-income countries (ILIC) and the average direct cost was compared by country of origin. A multivariate linear mixed model of direct costs was adjusted by country of origin (classified in five groups) and by the individual variables of age, gender, hospital admission, and death as a cause of discharge. Medical specialty was considered as a random effect.

**Results:**

With the exception of gynaecological emergency visits, costs resulting from emergency visits by both groups of immigrants were lower than those due to visits by the Spanish-born population. This effect was especially marked for emergency visits by adults.

**Conclusion:**

Immigrants tend to use the emergency department in preference to other health services. No differences were found between IHIC and ILIC, suggesting that this result was due to the ease of access to emergency services and to lack of knowledge about the country's health system rather than to poor health status resulting from immigrants' socioeconomic position. The use of costs as a variable of complexity represents an opportunistic use of a highly exhaustive registry, which is becoming ever more frequent in hospitals and which overcomes the lack of clinical information related to outpatient activity.

## Background

Several studies support the idea that immigrants make greater use of emergency services than other healthcare modalities and that the reasons for this phenomenon are diverse. Firstly, emergency care in Spain is public, free and universal, independently of nationality and length of residence [1]. In addition, primary and specialised care present several barriers to immigrants without papers [2-4]. Secondly, emergency care can be obtained without prior appointment and at any hour, facilitating compatibility with work schedules [5]. Thirdly, access to emergency care requires a small number of steps, thus reducing possible language, cultural and legal obstacles. Several studies report that differences in utilisation are reduced or even disappear when adjusted by gender, age, and socioeconomic position [6-8].

All of the above refers to a context of lower healthcare utilisation by the immigrant population than by the Spanish-born population. This is attributable to the "survivor effect", also known as the "healthy immigrant effect", according to which recently arrived immigrants have better health status than native-born residents [9-12]. This effect decreases with length of residence and the final result is an increased use of health services due to relative worsening of the immigrant's and better knowledge of how to access health services [10,13]. Other authors disagree with this concept and tend to believe that the immigrant population shows poorer self-perceived health and/or greater need and health services utilisation. These findings refer mainly to Nordic countries, in which there is a greater proportion of refugees from war-torn regions and fewer economic immigrants [7,14].

Currently, there are more than 15 million non-European immigrants living in Europe, the number and origin of these immigrants varying from country to country [15]. Spain has traditionally been a country of emigration and immigration is a relatively recent phenomenon. According to the **Statistical Office of the European Communities **[16]. Spain is the country in the European Union with the highest number of immigrants in 2005 in absolute terms, with 652,000 immigrants, representing most of the 1.7% (1.5%) of total population growth. Another characteristic of immigration to Spain is the country of origin, with a high percentage of immigrants from Latin America. The regions of Spain currently attracting the highest inflow of immigrants are Catalonia and Madrid [17]. The number of foreign residents in Catalonia increased from 60,800 in 1991 to 878,811 in 2005, representing 12.5% of the total population. Eighty percent of the immigrants in Catalonia are from low-income countries (ILIC) and of these, 61% are from Latin America, 20% from Asia, 11% from Africa and 8% from Eastern Europe. Only 1.6% of the ILIC population is aged above 65 years, while among the Spanish-born population this percentage is 27%. Spain does not have large collectives fleeing their countries of origin due to political reasons or natural disasters and therefore most ILIC are economic immigrants.

The present study distinguishes between immigrants from ILIC and those from high-income countries (IHIC). This distinction is made for several reasons: the legal status of citizens of the European Community is similar to that of the Spanish-born population and their socioeconomic status is above the national average [18][19].

Our hypothesis was that immigrants preferentially use the emergency department to access healthcare. As a result, emergency care in this population involves a lower workload than that corresponding to the Spanish-born population due to the lower level of complexity. Consequently, much of this care could be provided in primary care. In Spain, the private health sectors is small, accounting for approximately 15%, and, especially in emergency care, the vast majority of the population uses the public health services.

In Catalonia (Spain) information on patients' country of origin, case mix in emergency care and the costs attributable to these episodes is stored separately and is not easily related. Our hospital information sources allow case mix to be adjusted through specialty and variable costs (direct consumption) registered for each emergency visit. Currently, because the catchment areas fall within a metropolitan area, populational information is not available, since the various catchment areas overlap. Consequently, attendance rates cannot be analysed and the present study is based on analysis of utilisation.

In 2001 the hospital case mix of Hospital del Mar in Barcelona was analysed [20]. The wave of immigration intensified immediately before the above-mentioned study was performed. In 2000, the immigrant population represented 20% of the district of Ciutat Vella, which is within the catchment area of Hospital del Mar. By the end of 2005 this percentage had increased to 38.5%. Sixty-five percent of hospital discharges in immigrants were related to paediatrics and gynaecology, indicating that the profile of the immigrant user of hospital services corresponded to a young woman with higher fertility than that in the host population. For the remaining diagnostic groups, no substantial differences were found with respect to the Spanish-born population, although hospital services utilization among immigrants was low in view of the percentage of immigrants in the hospital's catchment area. Emergency visits (13%) were twice as frequent as admissions (7%) and three times as frequent as outpatient visits (4%).

Since then, further evidence has indicated that hospital services utilisation by the immigrant population is centered on paediatric and obstetric services.

We hypothesised that greater variable cost is related to higher complexity due to the greater workload involved in the diagnosis and follow-up of the patient.

The aim of the present study was to determine the workload generated by emergency department visits by immigrants according to country of origin.

## Methods

A total of 165,267 emergency visits to Hospital del Mar between 2002 and 2003 were included in the analysis. From 2001–2004 information was gathered on the country of origin of immigrants attending the emergency department of Hospital del Mar. The cost per patient in 2002–2003 was also calculated, thus enabling the workload involved in each emergency visit to be evaluated.

### Country of origin

The patients' country of origin was identified and the patients were subsequently divided into three large groups: Spanish-born residents, IHIC (European Union, Switzerland, Finland, the USA, Canada, Japan, Australia and New Zealand), and ILIC, which included all remaining countries. In turn, ILIC were divided into groups of countries according to geographical criteria: Latin America, northern Africa and the Nile Valley, sub-Saharan Africa, the rest of Asia, and eastern Europe. Country of origin was identified through the hospital's admissions registry. Patients from the department of and neonatology were classified according to the parents' country of origin.

### Specialty

Emergency care episodes were classified into five large clinical categories (obstetric and gynaecological, paediatric, medical, surgical, and traumatological), which, for the purposes of this study, are termed "specialties". The type of specialist who will attend the user is determined by initial triage in the emergency department.

### Cost

All hospital activity is costed under Activity-Based Costing criteria. Thus all discharges (admissions and ambulatory surgery), day hospital sessions, minor surgery, outpatient consultations and emergency visits are evaluated once the unit cost of each activity involved is known. For the hospital as a whole, the sum of all the final products expressed will correspond exactly to the total cost for the period analysed. When costing an emergency visit, two main activities are evaluated: on the one hand, the costs imputed to the emergency medical and nursing services, which are used to generate the unit costs of the physicians' and nursing staff's workload according to the time the patient spends in the emergency department. On the other hand, variable costs, which are those directly related to the patient according to the medical records and the unit costs corresponding to prostheses and the activities of laboratory, pathology, pharmacy and complementary tests, which effectively function as external companies and bill for each product and patient. The cost of an emergency visit, as analysed in the present study, did not incorporate the cost incurred if the patient was admitted to the hospital, given that the aim was only to compare the workload associated with visits to the emergency department.

Ethical approval was obtained from the Local Research Ethics Committee.

### Analysis

The average variable cost stratified by specialty, gender and age group (0–15, 16–50, 51–65 years and more than 65 years) and groups of country of origin (Spanish-born patients and IHIC versus ILIC) was calculated.

For each, gender-group, age group and specialty, the average cost was compared between the two groups of countries and Student's t-test for two independent samples was applied. Subsequently, the strata of immigrants incurring lower costs than Spanish-born residents, those incurring higher costs and those showing no differences with Spanish born residents were grouped together.

Lastly, a mixed linear model was adjusted with random effects in which direct cost variability was explained according to the patients' country of origin and was adjusted by gender, age, and death or hospital admission as causes of discharge from the emergency department. Specialty was considered as a random effect that was controlled for by adjusting the model. Both the dependent variable of variable cost and the explanatory variable of age were transformed logarithmically to normalise the variable of costs as far as possible and to be able to read the B parameter in terms of elasticity. Elasticity is the ratio of the incremental percentage change in cost with respect to an incremental percentage change in each dependent variable.

Most of the analysis was performed with the statistical package SPSS V11.5 and the mixed linear model was adjusted through SAS.

## Results

From 2002–2003 there were 165,257 emergency visits, of which 19.9% were made by immigrants. When visits made by ILIC only were included, this percentage was reduced to 15.5% (Table 1). Among Spanish-born residents, 38% of visits were made by persons aged more than 50 years old. This percentage varied among immigrants, depending on country of origin, but did not exceed 8% in ILIC and 13.7% in IHIC. In contrast, 78% of visits by immigrants were made by individuals aged 16–50 years old compared with 44% of those by Spanish-born residents. No substantial differences were found in individuals aged less than 16 years old.

**Table 1:** Distribution of age, gender, specialty and variable cost of emergency visits

	**Spanish-born residents**	**IHIC**	**Eastern Europe**	**Maghreb and Nile valley**	**Rest of Africa**	**Latin America**	**Asia**	**ILIC**	**Immigrants (IHIC + ILIC)**	**Total**
**Patients (n)**	132,435	7,226	1,587	5,020	681	13,776	4,532	**25,596**	**32,822**	**165,257**
**(%)**	80.1	4.4	1.0	3.0	0.4	8.3	2.7	**15.5**	**19.9**	**100.0**
**Age (%)**										
less than 16 years	16.8	10.8	11.2	11.5	15.3	16.4	16.9	**15.2**	**14.2**	**16.3**
between 16 and 50 years	44.2	75.6	84.6	80.7	78.3	77.8	77.9	**78.8**	**78.1**	**51.0**
between 51 and 65 years	12.2	7.9	3.1	5.3	6.2	3.8	4.2	**4.2**	**5.0**	**10.8**
more than 65 years	26.7	5.8	1.1	2.5	0.3	2.1	1.1	**1.9**	**2.7**	**21.9**
**Total**	100.0	100.0	100.0	100.0	100.0	100.0	100.0	**100.0**	**100.0**	**100.0**
										
**Gender (%)**										
Women	54.3	54.1	56.3	46.6	51.5	66.2	44.8	**57.5**	**56.8**	**54.8**
Men	45.7	45.9	43.7	53.4	48.5	33.8	55.2	**42.5**	**43.2**	**45.2**
**Total**	100.0	100.0	100.0	100.0	100.0	100.0	100.0	**100.0**	**100.0**	**100.0**
										
**Specialty (%)**										
Paediatrics	13.0	9.0	7.6	9.7	14.8	14.3	15.6	**13.2**	**12.3**	**12.8**
Gynaecology	11.5	17. 6	31.3	21.4	22.6	31.3	24.7	**28.0**	**25.7**	**14.3**
Surgery	15.2	16.0	14.6	15.7	12.3	10.9	13.8	**12.6**	**13.4**	**14.8**
Traumatology	22.2	23.3	14.1	21.5	13.4	15.6	13.6	**16.3**	**17.8**	**21.3**
Medicine	38.2	34.2	32.5	31.8	36.9	27.8	32.2	**29.9**	**30.8**	**36.7**
**Total**	100.0	100.0	100.0	100.0	100.0	100.0	100.0	**100.0**	**100.0**	**100.0**
										
**Variable cost**										
Total variable cost (€)	1,571,745	57,312	10,663	38,433	6,956	96,020	32,550	**184,622**	**241,935**	**1,813,680**
Total cost (%)	86.7	3.2	0.6	2.1	0.4	5.3	1.8	**10.2**	**13.3**	**100.0**
Mean variable cost	11.9	7.9	6.7	7.7	10.2	7.0	7.2	**7.2**	**7.4**	**11.0**
Standard deviation	35.2	24.7	17.9	22.8	38.3	22.2	22.6	**22.4**	**23.1**	**31.9**

ILIC = immigrants from low-income countries

IHIC = immigrants from high-income countries

Differences in gender were found by groups of countries, although no common pattern could be established among immigrants. Males predominated among immigrants from Asia and northern Africa, while females predominated among those from Latin America and sub-Saharan Africa.

Differences in specialty were found in greater relative utilisation of gynaecology and obstetrics services and lower utilisation of medicine and traumatology among ILIC compared with Spanish-born residents and IHIC.

The average variable cost was 10.97€ per visit. The average cost per emergency visit was 11.66€ for Spanish-born residents and IHIC, and was 7.21€ for ILIC.

**Figure 1:**
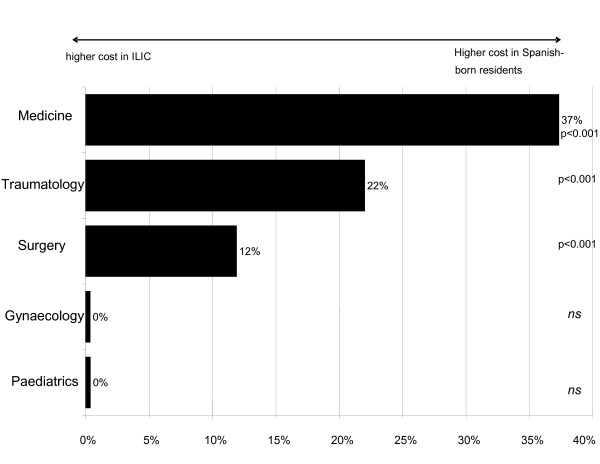
Deviation of the variable cost of Spanish-born residents with respect to ILIC.

Differences in average variable cost according to country of origin and specialty are shown in Figure 1. Variable cost was significantly lower for ILIC among the specialties of medicine (37%), traumatology (22%) and surgery (12%). No differences were found between obstetrics and gynaecology and paediatrics.

Because of the differences in age and gender between the two groups analysed (Spanish born residents and IHIC and ILIC), a stratified comparison was made (Table 2). The group aged 16–50 years, which was the largest age group among immigrants, showed significant differences in the expected direction (lower variable cost for ILIC) for the specialties of medicine and traumatology; however, these differences lost significance for the specialty of surgery, although the expected sign was maintained. These results were independent of gender. In contrast, for the same age group, utilisation among immigrants was significantly higher in gynaecology and obstetrics.

**Table 2:** Percentage of emergency visits by ILIC with costs showing significant differences (higher or lower)* from those made by Spanish-born residents + IHIC, grouped by gender, age and speciality.

	**Males**	**Females**	**Total**
***Lower resource consumption***			
Medicine	87.8	98.0	92.5
Traumatology	87.3	89.1	87.9
Surgery	0.0	6.6	2.2
Paediatrics	2.9	0.0	1.6

**Total Lower resource consumption**	**55.5**	**32.8**	**42.4**

***Higher resource consumption***			
Gynaecology	-	98.8	98.8

**Total Higher resource consumption**	**0.0**	**47.7**	**27.5**

*p < 0.05

ILIC = immigrants from low-income countries

IHIC = immigrants from high-income countries

When the distinct gender and age groups were classified according to whether differences were significant or not and, if significant, in which sense, 42.4% of emergency visits showed the expected association (lower cost among emergency visits by ILIC), 27.5% showed higher cost among emergency visits by ILIC, and 30.1% of the visits involved age groups in which the deviation was not significant.

Lastly, the characteristics that could explain variations in variable cost were analysed as a whole through a mixed linear model with logarithmic transformation of continuous variables (Table 3). The variables of the groups of country of origin were introduced as dichotomic variables for each category. Because it was the most numerous group, the Spanish-born population was taken as the reference category. The estimation presented an overall explanatory capacity (R^2^) of 9.1%. Notable in this model was the behaviour of all groups of countries of origin. Both IHIC and ILIC showed lower costs than did Spanish-born residents. Emergency variable cost was 9 to 17% lower for all immigrant groups. IHIC showed a negative deviation of 17.2%, quantitatively the most important.

**Table 3:** Multivariate adjustment (1) of the variable cost of emergency visits according to country of origin (2002–2003)

**Regression coefficient**	**Elasticity**	**(95% CI)**
ILIC from Maghreb and northern Africa	-0.1262	(-0.1664 to -0.0861)
ILIC from Latin America	-0.0967	(-0.1221 to -0.0714)
ILIC from Asia	-0.1502	(-0.1925 to 0.1079)
Other ILIC	-0.0897	(-0.1487 to -0.0308)
IHIC	-0.1717	(-0.2054 to -0.1380)
Age (ln)	0.1899	(0.1792 to 0.2006)
Gender (male)	-0.0473	(-0.0622 to -0.0324)
Death	1.9867	(1.8643 to 2.1091)
Hospital admission	0.1496	(0.0777 to 0.2215)
**Variance analysis**		
Intercept variable	0.1069	(0.0421 to 0.6200)
Individual variance	2.0109	(1.9972 to 2.0246)
Intra-speciality correlation	5%	
**Dependent variable: variable cost (ln transformation)**		
Cases analysed	165,257	
R2	9.1%	

(1) Mixed linear model of random effects for emergency specialty

ILIC = immigrants from low-income countries

IHIC = immigrants from high-income countries

The values of the adjustment variables were normal for age, with a positive elasticity of 19%. Gender showed little variation although male gender implied lower variable cost in emergency care, death as a reason for discharge showed an increased cost that almost tripled basal cost, and hospital admission carried a small increase in workload. Specialty was incorporated into the model as a random effect to control for its impact on the care provided but did not form part of the estimation of the model's parameters. Medicine and traumatology showed a higher cost independently of country of origin. Gynaecology showed lower cost. No significant differences were found in surgery or paediatrics with respect to the average for all emergency visits.

## Discussion

Differences in variable cost reflect lower complexity of emergency care due to the lower workload involved. The variable costs of a substantial number of emergency visits were analysed, enabling a series of distinguishing features to be established according to the patients' countries of origin. The results can be discussed according to the type of analysis performed; those presenting few discrepancies, or none at all, will be discussed first.

Emergency visits by adult immigrants (16–50 years) involving medicine, traumatology and surgery occasioned lower workload than those by Spanish-born residents and reductions in variable costs exceeded 10%. The remaining age groups were insignificant, as they included a small immigrant population. The results were fairly consistent for these three large specialties and can be generalised for all the age categories, given that the signs of the differences were homogeneous, although, due to the small number of cases, they did not reach statistical significance. Notably, the specialties of medicine and traumatology were those that most closely fitted the original hypothesis, since they showed higher variable costs and, therefore, their ability to act as a proxy of workload-complexity was greater.

Emergency visits involving gynaecology and paediatrics showed no overall differences. When stratified by age, higher variable cost was found in gynaecological emergencies for ILIC. This difference in cost was very low (9% of an average cost of 3.8€) and, although this difference was clearly statistically significant (p < 0.008), its economic importance was lower than in other specialties, in which the reduction in cost was much higher in percentage and absolute values.

The expected finding of greater emergency department use services utilisation among immigrants than among Spanish-born residents was consistent among the adult population, supporting the hypothesis that immigrants overcome certain barriers by using the emergency department as the route of access to health services in preference to other routes. The finding that IHIC also showed greater emergency services utilisation department use suggests that the cause lies in overcoming barriers to access rather than in immigrants' socioeconomic position. Furthermore, the average income in both the immigrant and the native populations within the hospital catchment area is low. Since both IHIC and ILIC showed the same behaviour, the most coherent explanation seems to be the short length of residence among immigrants and their consequent lack of knowledge of the normal routes of access to health services.

The finding that gynaecological emergencies in adult immigrant women of reproductive age represented greater workload than those by their Spanish-born counterparts can be explained by the difficulties of pregnancy follow-up in ILIC in primary care. Participation in primary care pregnancy follow-up programs is substituted by sporadic contact with the emergency services that perform the pertinent procedures, with the variable cost that this represents. The same occurs at delivery, increasing the cost of hospital care of the neonate in the early postpartum period [21].

Importantly, socioeconomic position and health status in the catchment area as a whole are lower than the averages for the city of Barcelona. This is an important difference and the differences detected may be smaller than those that would have been found if immigrants had been compared with a Spanish-born population with better health, social and economic indicators.

This study presents several limitations. Firstly, variable cost is used as an approximation of workload and, in turn, this workload is what determines whether the emergency visit constitutes a real emergency or could have been treated in primary care. This is undoubtedly an indirect approximation. However, publications that provide direct evidence in our environment are lacking. The methodology used in this study is therefore conceived of as an alternative that may provide information useful for adapting hospital services to the non-Spanish-born population and for indicating the interventions required to achieve utilisation patterns similar to those of the Spanish-born population.

A second limitation concerns a possible subregister of immigrant patients, basically of children born in Spain who, to all intents and purposes, count as Spaniards; what matters in our analysis is behaviour relating to the socioeconomic and cultural position of the family and, in this sense, these children are immigrants. The paediatric and neonatology database constitutes an important attempt to minimise this problem; since 2001 the parents' country of origin has been registered, representing a significant improvement. Even so, a certain subregister of these children who were not born in or admitted to the hospital, or who did so before 2001, may still exist in the emergency department.

A third limitation concerns the profile of utilisation among the visits that took place, which cannot be used to infer populational behaviour. This problem could be resolved by determining the origin of all the patients attending the emergency department of the hospitals in a sufficiently large area to ensure that all the emergency visits by a specific population are included. This is a methodological alternative that implies the use of the variable of cost as an indicator of complexity and which, by itself, has demonstrated its viability.

The fourth limitation is due to the scarcity of clinical information available on emergency visits. This is a consequence of the policy in the Spanish health system, which has prioritised codification of hospital admissions, as this was an essential step in constructing diagnosis-related groups (DRGs). An analogous system to DRGs has not been established for ambulatory activities, which has discouraged codification of ambulatory activity. This is a general defect, affecting the entire system, and should be resolved; however, until this happens, the available information must be used. In this sense, differentiation by specialty can be useful, as shown in the present study.

Notable among the strengths of the study is determination of the variable costs registered for all the emergency visits and their association with country of origin. This information allows an analysis that provides a certain level of evidence that is useful for the planning of health services and interventions to improve the healthcare of the distinct emerging collectives in the Spanish health system.

## Conclusion

Leaving aside the behaviour of the profile of gynaecological and paediatric emergency visits from the remaining emergency visits among adults, some useful conclusions for health policy can be drawn. The immigrant population tends to access the health service through the emergency department even more than the Spanish-born population. Consequently, the substantial increase in both IHIC and ILIC in the health system has a multiplying effect on the lack of equilibrium that an increase in demand for emergency services represents for a hospital. The increase in pressure on the emergency services is probably related to lower levels of efficiency, since it involves the use of expensive high-intensity resources to respond to non-urgent conditions that could be managed in the primary care setting [5].

Because of the relatively high volume of obstetric and gynaecological emergency visits, as well as their level of complexity, greater efforts should be made to reach immigrant women of reproductive age and include them in antenatal care programs.

To take the next step in investigating the impact of the immigrant population on the Spanish health system, a direct relationship with the populational setting should be established, to characterize not only patterns of use but also attendance rates.

## Competing interests

The author(s) declare that they have no competing interests.

## Authors' contributions

All the authors have contributed to the achievement of this study. FC conceived of the study, participated in the design of the study, performed the main statistical analysis and drafted the manuscript. XC, OV and OG participated in the design of the study and helped to draft and review the manuscript. AF and MR performed data management and review the manuscript. All authors read and approved the final manuscript.

## Pre-publication history

The pre-publication history for this paper can be accessed here:


